# *Hermetia illucens*-Derived Chitosan: A Promising Immunomodulatory Agent for Applications in Biomedical Fields

**DOI:** 10.1021/acs.biomac.5c00362

**Published:** 2025-04-29

**Authors:** Alessandra Fusco, Anna Guarnieri, Carmen Scieuzo, Micaela Triunfo, Rosanna Salvia, Giovanna Donnarumma, Patrizia Falabella

**Affiliations:** † Department of Life Sciences, Health and Health Professions, Link Campus University, 00165 Rome, Italy; ‡ Department of Experimental Medicine, University of Campania “Luigi Vanvitelli”, Naples 80138, Italy; § Department of Basic and Applied Sciences, University of Basilicata, Via dell’Ateneo Lucano 10, 85100 Potenza, Italy; ∥ Spinoff XFlies s.r.l, University of Basilicata, Via dell’Ateneo Lucano 10, 85100 Potenza, Italy

## Abstract

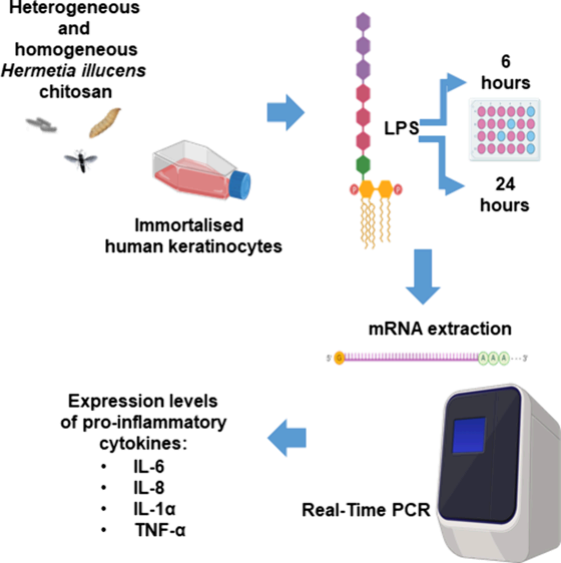

Chitosan, renowned for its important biological properties, is a valuable pharmaceutical excipient for different therapeutic approaches. Currently, the demand for the biopolymer on the market is growing, and, for this reason, it is important to biologically characterize the biopolymer produced from an alternative source to crustaceans, specifically the bioconverter insect *Hermetia illucens*. In this work, insect chitosan, yielded via heterogeneous and homogeneous deacetylation from larvae, pupal exuviae, and adults, was studied as an immunomodulatory agent. The inflammatory response of immortalized human keratinocyte cells was induced by *Salmonella enterica* subsp. *enterica* serovar Typhimurium lipopolysaccharide. After that, the ability of the biopolymer to reduce the expression of the pro-inflammatory cytokines IL-6, IL-8, IL-1α, and TNF-α was tested after 6 and 24 h of treatment. Insect chitosan samples effectively downregulated cytokine expression, with improved activity obtained from heterogeneous chitosan treatments.

## Introduction

The inflammatory response system represents the primary defense mechanism of the human and mammalian body against infections caused by a variety of harmful stimuli, such as pathogens, damaged cells, toxic compounds, or irradiation.^1^ Heat, redness, swelling, pain, and tissue function loss are the features of inflammatory cell responses at the tissue level.^2^ In order to counteract inflammatory processes, research has focused on designing composite systems that release active substances at the inflamed site, neglecting the anti-inflammatory potential of any single excipient used as it is. These might have a direct therapeutic effect, in addition to their role in specific composite formulations. The use of chitosan is an outstanding instance of this strategy.^3^

As a polysaccharide of natural origin, chitosan is renowned for its high biocompatibility and proven safety profile.^4,5^

Besides that, this biopolymer has important properties related to tissue regeneration, such as not only the ability to promote angiogenesis and stimulate collagen synthesis but also antimicrobial and anti-inflammatory activity.^6−11^

There are few studies in the literature regarding the anti-inflammatory activity of chitosan. Among these, some researchers have investigated the anti-inflammatory activity of chitosan oligosaccharides on mouse models with osteoarthritis and allergic asthma, noting a reduction in inflammatory cytokines.^12,13^

Currently, because of its wide range of applications, the market for chitin, the nondeacetylated chitosan precursor, is worth millions of dollars.^14^ However, seeking alternatives to crustaceans, the commercial source of these biopolymers,^15^ that can supply their demand, paves the way for new commercial opportunities, especially in the field of personal health.

The availability of fishing supplies is affected both by seasonal and geographical limitations,^16^ while insects are a viable and more sustainable alternative source of the two biopolymers.^15,17^ Insect farming requires fewer resources—such as land, water, and feed—compared to traditional aquaculture.^18^

In addition, some insects, particularly *Hermetia illucens* larvae, are able to bioconvert waste products from the agri-food chain.^19−23^ Chitin can be extracted from all developmental stages of *H. illucens*.^24,25^ In this way, insect breeding ensures a continuous supply of the biomasses from which the biopolymers can be extracted, and allows for the valorization of waste biomass from the breeding process itself, such as pupal exuviae and adults, which are rich in chitin.^26^

Its production allows for extremely advantageous ecological, economic, and environmental supply and impact, and can guarantee constant chitosan production that meets all market requirements, not possible with crustacean processing. For all these reasons, *H. illucens* breeding is embedded in a zero-waste circular economy system.^27^

Insect chitosan, produced by both heterogeneous and homogeneous deacetylation,^17,28^ has shown comparable or even better biological properties than the crustacean biopolymer, such as antioxidant, antimicrobial, and antifungal activities.^29−31^

Furthermore, chitosan, known for its bioactivity and biocompatibility, can interact with cells via cell surface receptor ligands, for example, in the form of electrospun structures.^32−36^ Keratinocytes are the main cellular components of the skin. In case of injury, these cells proliferate and differentiate to form the neoepidermis, restoring epidermal barrier function.^37^ Their proliferative capacity is crucial to facilitate interaction with the other cell types involved in this process.^38^

Chitosan can be defined as a biomimetic polymer due to its hemostatic, healing-stimulating, and biodegradable properties that make it suitable for effective use in cutaneous tissue regeneration.^39−41^ Native extracellular matrix possesses glycosaminoglycan (GAG), consisting of the N-acetylglucosamine that is also included in chitosan. Since GAG allows specific interactions with cells, some studies have suggested that combining the biopolymer with other substances (e.g., poly(vinyl alcohol)) may improve cell proliferation and differentiation in skin regeneration processes.^42^

Among the most studied cells for this purpose, in order also to assess the effects of substances on skin systems there are HaCaT cells.^43^ They are derived from keratinocytes of dermal origin, and are immortalized but not tumorigenic,^44,45^ representing a trustworthy comparative model to normal human skin keratinocytes.^46^

Chitosan influence on HaCaT cells may be dependent on several factors such as acetylation degree,^47−49^ concentration, molecular weight, and incubation time. The mechanism of interaction between chitosan and keratinocytes is under debate^50^ but, surely, it has some effects on cytokine secretion.^51^

Proteins belonging to the cytokine family are responsible for intracellular signaling, creating and conditioning immune system responses.^52^ Cytokines, heterogeneous signaling molecules, play crucial roles in immune and inflammatory reactions, and in the regulation of cell growth and differentiation.^53^ The main types include interleukins (ILs), tumor necrosis factor (TNF), interferons (IFNs), chemokines, colony-stimulating factors (CSFs), and growth factors, all with specific functions.^54^

Maintenance of tissue homeostasis is related to the balance between anti-inflammatory and pro-inflammatory cytokines, whose alteration can result in many immunopathologies. Anti-inflammatory cytokines perform a pivotal function in regulating and resolving inflammation.^55^ On the contrary, pro-inflammatory cytokines amplify the immune response, and they can exacerbate diseases by promoting systemic inflammation. They are involved in triggering inflammatory reactions, and they are mainly produced from activated macrophages, immune system cells.^56^

IL-1 is a cytokine produced by various cell types, such as endothelial cells and keratinocytes, and can be found in two forms (IL-1α and IL-1β). Despite sharing only 30% structural homology, these forms have nearly identical biological activities.^57^ At low doses, IL-1 stimulates local inflammation and coagulation, while at higher doses, it acts as an endogenous pyrogen, inducing the production of acute-phase proteins and potentially leading to cachexia.^57^

TNF-α is a key component of the inflammatory cascade.^58^ When released in the inflammation area, TNF-α activates the vascular endothelium in that specific region, leading to the release of nitric oxide (NO). It causes vasodilation, facilitating the influx of inflammatory cells, immunoglobulins, and the complement system to the site of injury. Additionally, TNF-α is involved in coagulation by influencing platelet adhesiveness and contributing to thrombus formation and vascular occlusion, thereby limiting the spread of infection but also increasing the risk of tissue necrosis.^59^

IL-6 is a multifunctional molecule that acts as a mediator of inflammation in its autocrine, paracrine, and endocrine roles.^60^ Among its main functions, there is the stimulation of hepatocytes to produce numerous blood proteins, including fibrinogen, which are essential for the acute inflammatory response.^61^

IL-8 is secreted by a variety of cells, including monocytes, macrophages, fibroblasts, endothelial cells, and keratinocytes.^62^ It plays a crucial role in attracting and activating neutrophilic leukocytes, promoting neutrophils, basophils, and T lymphocytes migration.^63,64^ In addition to these, IL-8 has other functions, such as guiding the movement of basophils and contributing to the formation of new blood vessels (angiogenesis).^65^ When injected into the skin, IL-8 causes an immediate inflammatory reaction, evidenced by the rapid influx of neutrophils into the affected area.^66^

The aim of this work was to test, for the first time, the anti-inflammatory activity of chitosan, both heterogeneously and homogeneously deacetylated, produced from pupal exuviae, larvae, and adults of *H. illucens*, on immortalized human keratinocytes (HaCat cells), in order to further characterize the biopolymer and then validate its use in biomedical and pharmaceutical fields.

## Methodology

### Cell Culture

HaCat cells were cultivated in culture medium consisting of Dulbecco’s Modified Eagle Medium (DMEM). This medium was enhanced with a 10% volume/volume (v/v) addition of Fetal Bovine Serum (FBS) and further supplemented with 1% Penstrep (100 U/ml penicillin and 100 μg/mL streptomycin) and 1% l-glutamine. The cells were maintained at 37 °C in a humidified incubator that provided a 5% CO_2_ atmosphere. For performing the experiments, HaCat cells were placed in 24-well plates. They were cultivated until they reached 80% confluence, indicating dense but not overcrowded cell growth.

### Heterogeneous and Homogeneous Chitosan Production

Raw insects, specifically pupal exuviae, larvae, and adults of *H. illucens*, were provided from Xflies s.r.l (Potenza, Italy).

Unbleached and bleached heterogeneous chitosan samples from the three biomasses were obtained following the method described in Triunfo et al.,^17^ while unbleached and bleached homogeneous samples were produced following the procedure reported in Triunfo et al.^28^

Chitosan sample identity was investigated by chemical-physical evaluation, particularly through Fourier transform infrared spectroscopy (FTIR) and X-ray diffraction (XRD).

Molecular weight (Mw) of heterogeneous chitosan samples ranged between 21 and 92 kDa, while the degree of deacetylation (DD) was around 90% for all the samples, slightly lower for unbleached pupal exuviae (83%).^17^*H. illucens* homogeneous biopolymer (from all three biomasses, both bleached and unbleached), on the other hand, featured higher Mw values (within 89–285 kDa) and lower deacetylation degrees (from 56 to 72%)^29^ (Table 1).

**Table 1:** Molecular Weight (Mw) and Deacetylation Degree (DD) of Heterogeneous and Homogeneous Chitosan Obtained from Both Unbleached and Bleached Chitin from *H. illucens* Larvae, Pupal Exuviae and Adults^17,28^

	chitosan sample	Mw (kDa)	DD (%)
heterogeneous	unbleached larvae	92	91
	bleached larvae	21	92
	unbleached pupal exuviae	55	83
	bleached pupal exuviae	35	90
	unbleached adults	62	91
	bleached adults	36	93
homogeneous	unbleached larvae	195	56
	bleached larvae	97	60
	unbleached pupal exuviae	285	62
	bleached pupal exuviae	115	72
	unbleached adults	258	59
	bleached adults	89	61

### Chitosan Solution Preparation and Cell Treatments

Unbleached and bleached chitosan samples from *H. illucens* larvae, pupal exuviae, and adults were dissolved in 17 mM CH_3_COOH,^50^ at a 10X final concentration; pH was adjusted to physiological point (7.0), and the solutions thus obtained were then sterilized with a 0.22 μm filter membrane (Euroclone-Primo Syringe Filters). During the assays, these solutions were diluted in DMEM to achieve a final concentration of 500 μg/mL. To assess cell viability, semiconfluent cells were exposed to chitosan-diluted solutions for 24 h. Cell inflammatory response was induced by *Salmonella enterica* serovar Typhimurium lipopolysaccharide (LPS), at a concentration of 20 μg/mL. After that, chitosan samples were added, and cells were incubated for 6 and 24 h.^67^ Additionally, untreated controls (one without any of the treatments and another treated exclusively with LPS) were kept to provide a baseline for comparison.

### Assessment of Cell Viability

Before the evaluation of the anti-inflammatory activity of *H. illucens* chitosan samples, the Alamar Blue assay was carried out. In order to assess the cytotoxicity of the different solutions of bleached and unbleached chitosan from *H. illucens*, HaCat cells were multi-well seeded and treated with the solutions at a concentration of 500 μg/mL. After 24 h of incubation, a resazurin solution at 500 μg/mL concentration was added to each well. The cells were subsequently incubated for a further 4 h at 37 °C. Commercial chitosan was used as a control (Sigma-Aldrich, St. louis-Missouri, USA).

In the study conducted, the metabolic activity of the cells was determined by measuring the absorbance at wavelengths of 570 and 600 nm, using a spectrophotometer. The results were quantified in terms of the percentage of reduced Alamar blue (%ABred), an indirect measure of cell viability. To calculate %ABred, the absorbance values obtained were correlated to the molar extinction coefficients of the compound. The following formula was applied for this calculation, including dual wavelength readings, allowing a more accurate estimate of the color change:
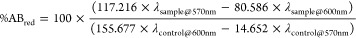


### Evaluation of *H. illucens* Chitosan Anti-Inflammatory Activity

#### RNA Extraction and qPCR

HaCat cells were treated with chitosan samples, either with or without *S. typhimurium* LPS for 6 and 24 h. Following the treatment, cells were lysed for mRNA extraction using the TRIzol reagent according to the manufacturer's protocol. The extracted mRNA was employed to synthesize complementary DNA (cDNA), using reverse transcriptase enzyme (Promega).

The resulting cDNA was used as a template for quantitative real-time PCR to evaluate the expression levels of pro-inflammatory genes IL-6, IL-8, IL-1α, and TNF-α.

Real-time PCR was carried out using the LC Fast DNA Master SYBR Green Kit (Roche Diagnostics) on a LightCycler 2.0 Instrument, according to the manufacturer protocols.

In Table 2, the primers used for qPCR are reported.

**Table 2:** Primers Used for qRT-PCR of IL-1α, TNF-α, IL-6, and IL-8

gene	primers sequences	conditions	amplicon size (bp)
*IL-1*α	F: 5′-CATGTCAAATTTCACTGCTTCATCC-3′	5″ at 95 °C, 8″ at 55 °C,	421
	R: 5′-GTCTCTGAATCAGAAATCCTTCTATC-3′	17″ at 72 °C for 45 cycles	
*TNF*-α	F: 5′-CAGAGGGAAGAGTTCCCCAG-3′	5″ at 95 °C, 6″ at 57 °C,	324
	R: 5′-CCTTGGTCTGGTAGGAGACG-3′	13″ at 72 °C for 40 cycles	
*IL-6*	F: 5′-ATGAACTCCTTCTCCACAAGCGC-3′	5″ at 95 °C, 13″ at 56 °C,	628
	R: 5′-GAAGAGCCCTCAGGCTGGACTG-3′	25″ at 72 °C for 40 cycles	
*IL-8*	F: 5-ATGACTTCCAAGCTGGCCGTG-3′	5″ at 94 °C, 6″ at 55 °C,	297
	R: 5-TGAATTCTCAGCCCTCTTCAAAAACTTCTC-3′	12″ at 72 °C for 40 cycles	
β-*actin*	F: 5′-GACGACGACAAGATAGCCTAGCAGCTATGAGGATC-3′		243
	R: 5′- GAGGAGAAGCCCGGTTAACTTCCGCAGCATTTTGCGCCA-3′		

F: Forward, R: reverse.

After each amplification cycle, a melting curve analysis was performed to ensure the absence of nonspecific amplification products. The accuracy of RNA quantification depends on the linearity and amplification efficiency of the PCR.

These parameters were assessed through the use of standard curves obtained by increasing cDNA amounts. RNA quantification employs cycle cutoff values measured during the onset of the exponential phase of the PCR reaction. In addition, a normalization is performed against a standard curve, obtained using actin as a housekeeping gene, to assess any mismatches in RNA input or transcription efficiencies.^67^

#### ELISA Assay

HaCat cells were treated with chitosan samples, with or without *S. typhimurium* LPS, as previously described, for 48 h. At the end of this time, the presence of IL-6, IL-8, IL-1α, and TNF-α in cellular supernatants was analyzed using enzyme-linked immunosorbent assay (ELISA; Elabscience Biotechnology Inc.; Phoenix Pharmaceuticals, Inc.).

## Results

### Heterogeneous and Homogeneous Chitosan Production

As shown in Figures S1 and S2, FTIR and XRD analysis of *H. illucens* chitosan samples confirmed insect biopolymer identity.^17,28^ Both heterogeneous and homogeneous chitosan samples yielded spectra comparable to that obtained from commercial chitosan, always employed as the experimental control. The results proved the identity of all insect biopolymers, featuring the typical profile of the two chitosan deacetylations, for both FTIR and XRD analysis

### Assessment of Cell Viability

The results of the Alamar Blue assay performed on the HaCat cells (Table 3) showed the cell viability in a very high range (assimilated to almost 100%) for all tested chitosan samples from *H. illucens*, both unbleached and bleached.

**Table 3:** Percentage ABred Values of Unbleached and Bleached Chitosan Samples Obtained from *H. Illucens* Larvae, Pupal Exuviae, and Adults and the Commercial One Derived from Crustaceans^a^

chitosan sample		% AB_RED_
heterogeneous	unbleached larvae	97 ± 5
	bleached larvae	97 ± 5
	unbleached pupal exuviae	99 ± 5
	bleached pupal exuviae	100 ± 5
	unbleached adults	102 ± 5
	bleached adults	96 ± 5
homogeneous	unbleached larvae	98.5 ± 5
	bleached larvae	101 ± 5
	unbleached pupal exuviae	94 ± 5
	bleached pupal exuviae	99 ± 5
	unbleached adults	96.3 ± 5
	bleached adults	97 ± 5
	commercial	99 ± 5

a Data are expressed as mean ± standard deviation. No significant differences were found among samples (data analyzed with one-way ANOVA and Tuckey *post-hoc* test).

### Evaluation of *H. illucens* Chitosan Anti-Inflammatory Activity

Chitosan samples (500 μg/mL) were tested for their ability to reduce the expression of the pro-inflammatory cytokines IL-6, IL-8, IL-1α, and TNF-α, induced by 20 μg/mL LPS on HaCat cells.

**Figure 1:**
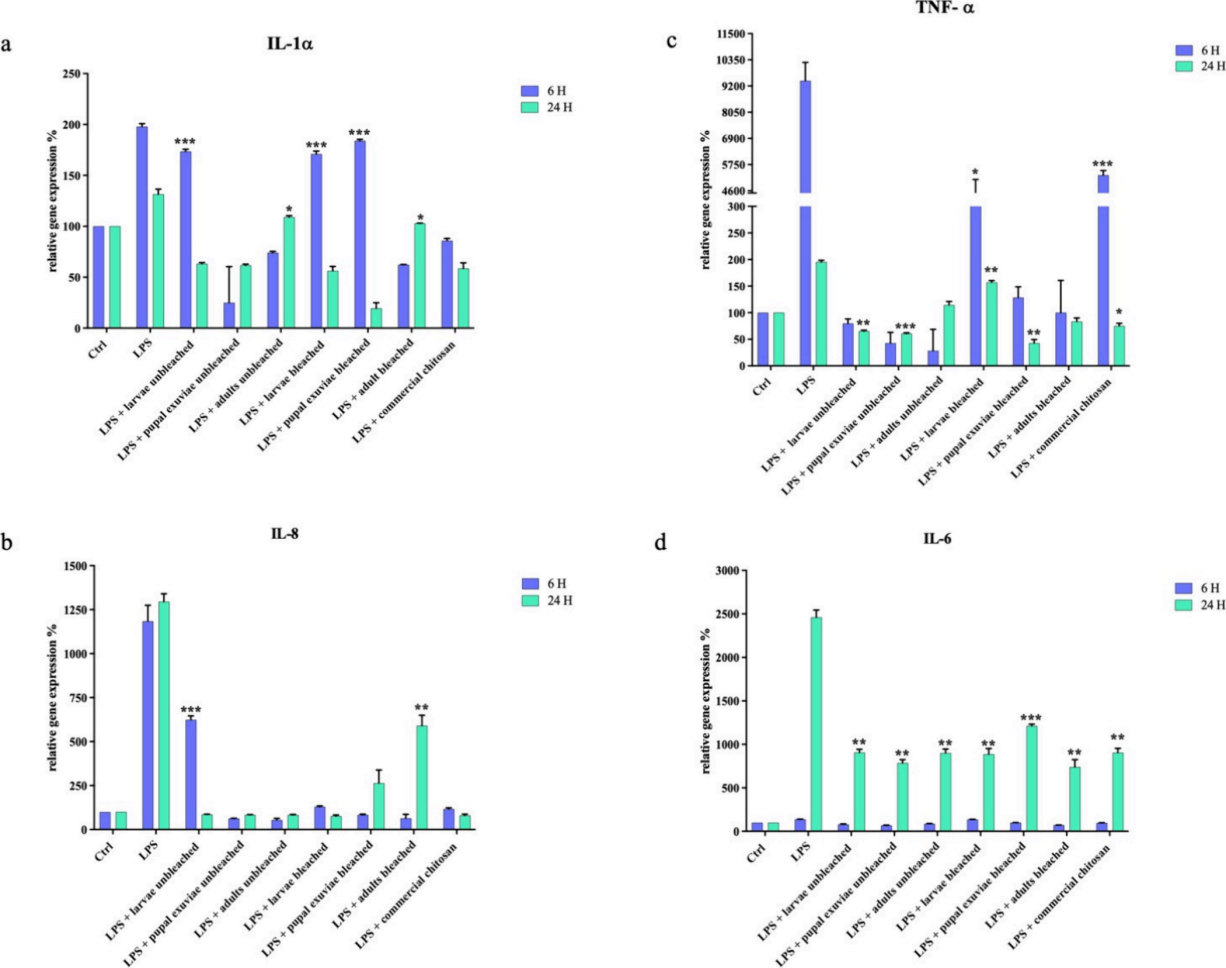
qPCR showed the expression levels of pro-inflammatory cytokines IL-1-α (a), IL-8 (b), TNF-α (c), and IL-6 (d) on HaCat cells treated with LPS and *H. illucens* heterogeneous chitosan samples, both bleached and unbleached. Data are mean ± SD and are expressed as a percentage of the relative mRNAs compared to unstimulated control (ctrl), arbitrarily assigned as 100%. Significant differences are indicated by *p < 0,05, **p < 0,01, ***p < 0,001. Data were analyzed with two-way ANOVA and Bonferroni *post-hoc* test.

**Figure 2:**
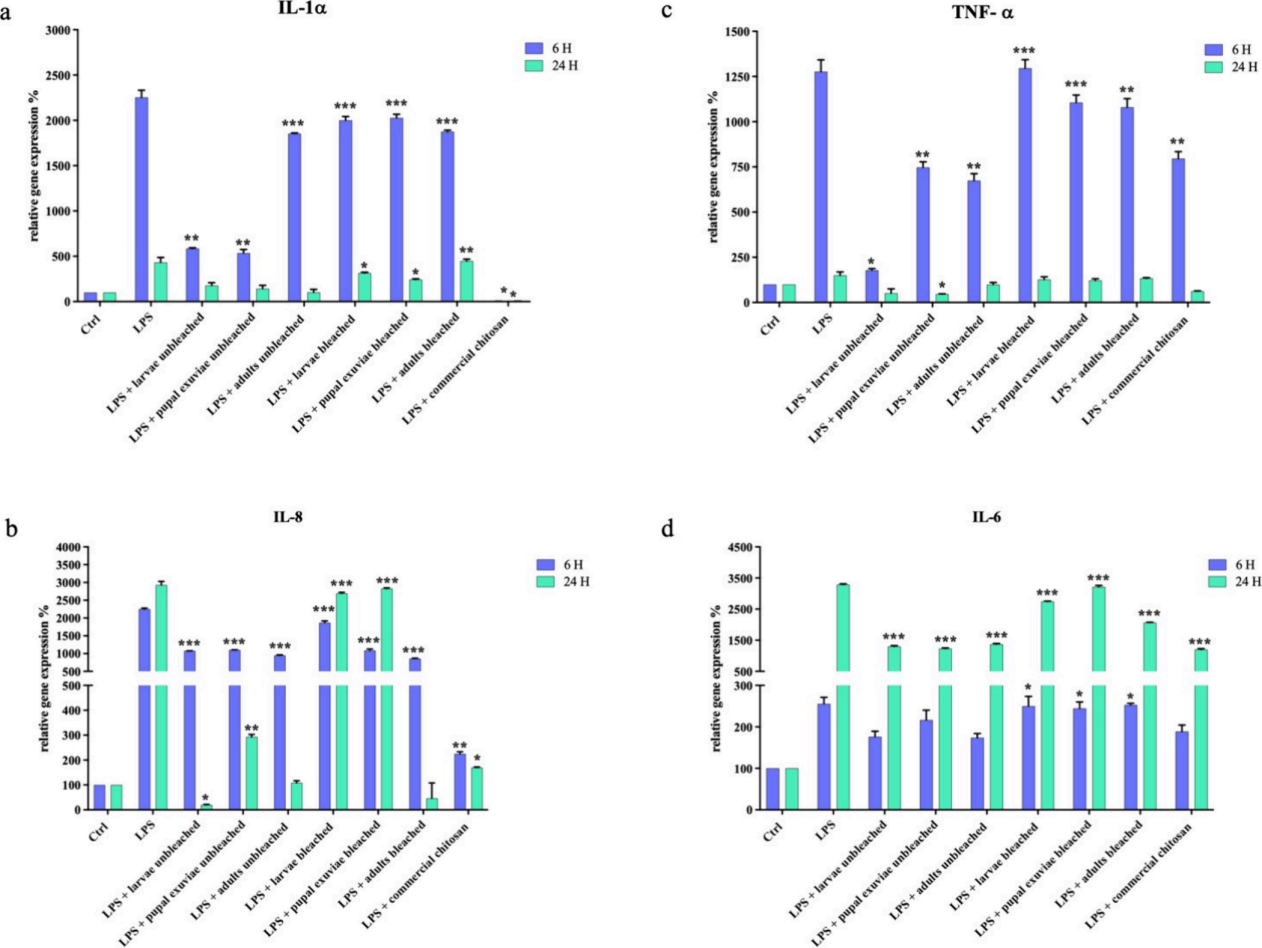
qPCR showed the expression levels of pro-inflammatory cytokines IL-1-α (a), IL-8 (b), TNF-α (c), and IL-6 (d) on HaCat cells treated with LPS and *H. illucens* homogeneous chitosan samples, both bleached and unbleached. Data are mean ± SD and are expressed as a percentage of the relative mRNAs compared to unstimulated control (ctrl), arbitrarily assigned as 100%. Significant differences are indicated by *p < 0,05, **p < 0,01, ***p < 0,001. Data were analyzed with two-way ANOVA and Bonferroni *post-hoc* test.

The qPCR results obtained, deriving from three independent experiments, expressed as the effective concentration in nanograms of amplified mRNA, showed that *H. illucens* heterogeneous chitosan produced from, at high concentrations, had a strong anti-inflammatory activity against LPS-treated cells. Indeed, the expression of all pro-inflammatory cytokines tested was significantly reduced (Figures 1 and 2). Although there were differences in expression between the different samples, especially between unbleached and bleached ones, generally homogeneous chitosan from *H. illucens* exhibited good anti-inflammatory capacity.

IL-1α was expressed as early as 6 h after treatments with LPS. It appeared to be significantly modulated by all heterogeneous chitosan samples, except bleached and unbleached chitosan from larvae and the bleached one derived from pupal exuviae, which showed mild modulation. The inhibitory effect was maintained by all samples even at 24 h of treatment, where, naturally, cytokine expression was reduced. As can be seen in Figure 1c, homogeneous chitosan samples from bleached insects did not give a significant modulating effect of IL-1α, whereas samples from unbleached larvae and unbleached pupal exuviae showed a very good decrease in the expression of this cytokine. These same samples also slightly modulated interleukin expression 24 h after treatment.

IL-8, an early response interleukin, was expressed already after 6 h of LPS induction, and it was maintained up to 24 h. It was found to be significantly modulated by all heterogeneous chitosan samples, among which the unbleached sample obtained from larvae and the bleached one derived from adults showed a lower effect at 6 and 24 h of treatment, respectively. Concerning homogeneous chitosan, after the first hour, all unbleached samples effectively modulated cytokine expression. Among the bleached ones, however, the best effect was achieved from adult chitosan-mediated modulation. Even at 24 h, all unbleached samples powerfully modulated IL-8 expression, while among the bleached samples, as was already the case at 6 h after treatment, adult chitosan was the most effective sample in its series.

TNF-α induction occurs similarly to the last two cytokines described above, after 6 h of treatment, as it is an acute-phase cytokine. All heterogeneous chitosan samples from *H. illucens* significantly reduced their expression, although there was less modulation by bleached chitosan from larvae. Commercial chitosan also yielded a low modulation of the cytokine. Homogeneous bleached chitosan samples did not appear to have a modulating effect on its expression of this cytokine, except in adults, which showed a slight effect. On the other hand, unbleached samples all effectively modulated TNF-α expression at 6 h of treatment, with a particularly strong effect on unbleached chitosan from larvae. At 24 h of treatment, the samples that showed persistently slightly lower cytokine expression were unbleached pupal exuviae and unbleached larvae.

**Figure 3:**
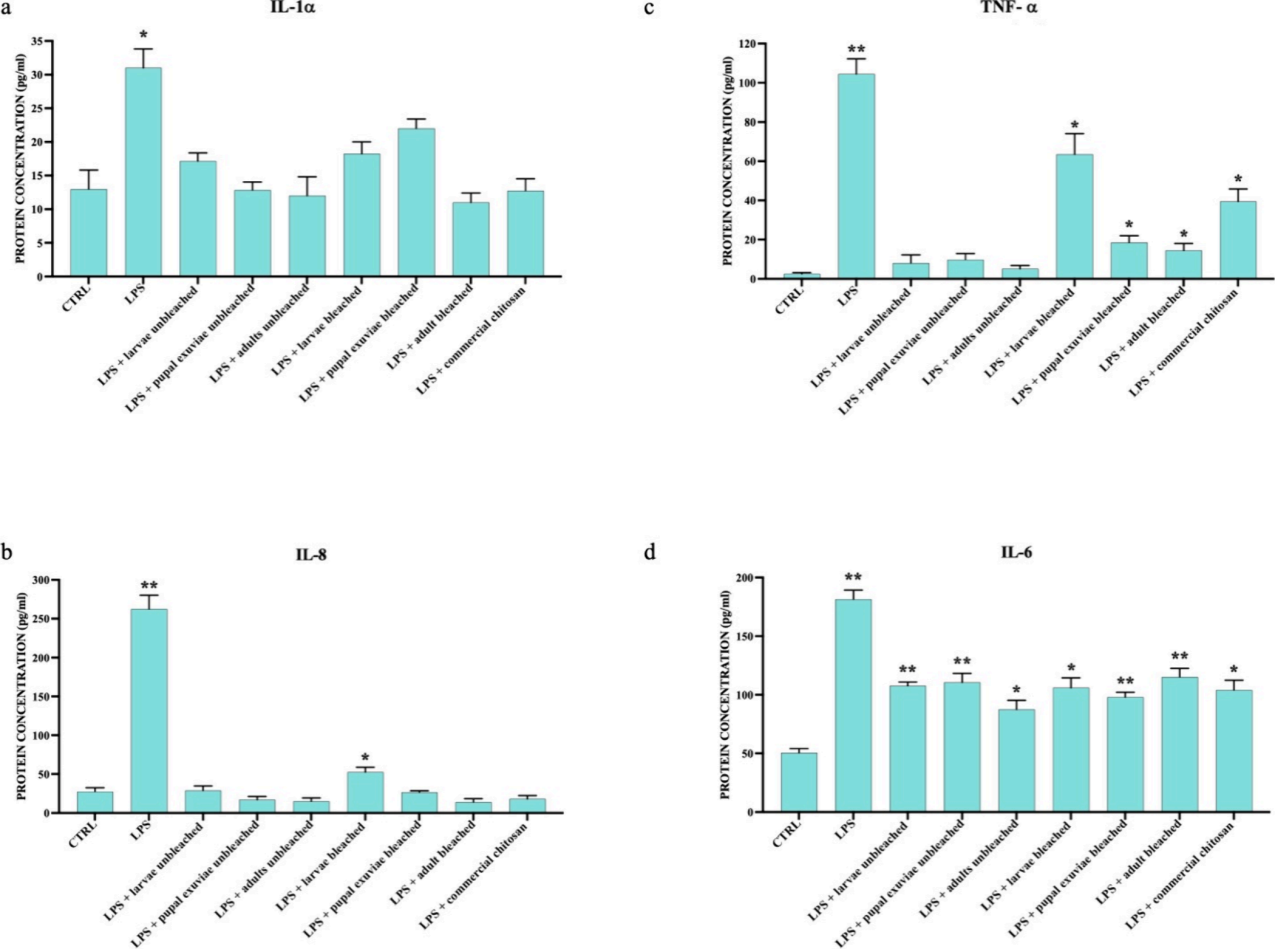
ELISA assay showed the concentration of pro-inflammatory cytokines IL-1-α (a), IL-8 (b), TNF-α (c), and IL-6 secreted in cell supernatants of HaCat cells treated with LPS and *H. illucens* heterogeneous chitosan samples, both bleached and unbleached. Data are expressed as pg/mL of protein concentration ± standard deviation in each group and are representative of three different experiments. Significant differences are indicated by *p < 0,05, **p < 0,01. Data were analyzed with two-way ANOVA and Bonferroni *post-hoc* test.

**Figure 4:**
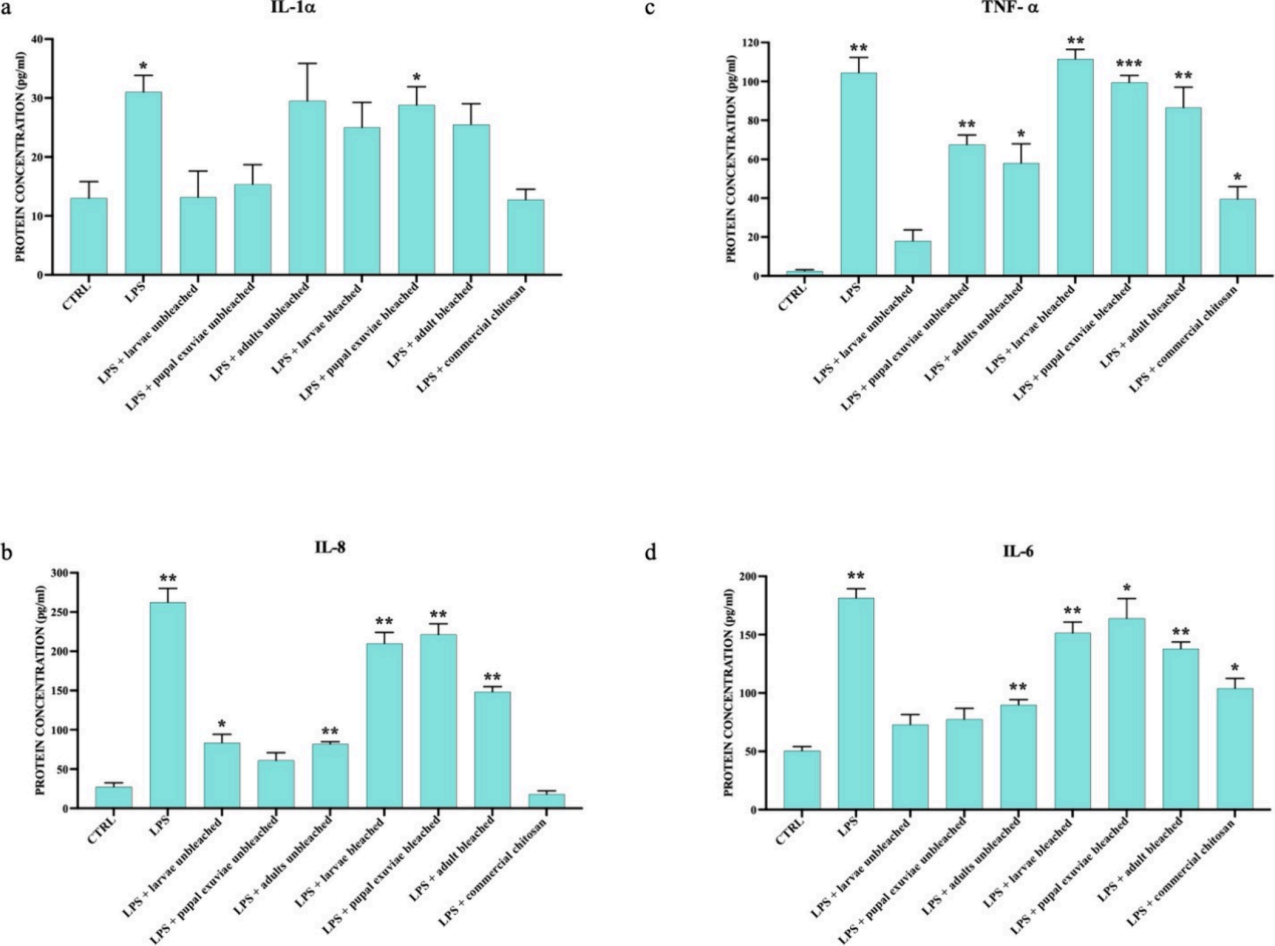
ELISA assay showed the concentration of pro-inflammatory cytokines IL-1-α (a), IL-8 (b), TNF-α (c), and IL-6 secreted in cell supernatants of HaCat cells treated with LPS and *H. illucens* homogeneous chitosan samples, both bleached and unbleached. Data are expressed as pg/mL of protein concentration ± standard deviation in each group and are representative of three different experiments. Significant differences are indicated by *p < 0,05, **p < 0,01, ***p < 0,001. Data were analyzed with two-way ANOVA and Bonferroni *post-hoc* test.

IL-6, unlike the previous mediators, occurred effectively after 24 h of LPS-induced treatment of the cells. It was modulated similarly by all heterogeneous chitosan samples, with a slight minor effect provided by bleached chitosan obtained from pupal exuviae. Unbleached homogeneous chitosan samples, from all three biomasses of *H. illucens*, were excellent modulators in downregulating the expression of this cytokine; among the bleached samples, however, chitosan from larvae and from adults gave the most effective modulation. These results were confirmed at the protein level by ELISA assay (Figures 3 and 4).

## Discussion

Both heterogeneous and homogeneous chitosan samples produced from larvae, pupal exuviae, and adults of *H. illucens* that were tested proved to be nontoxic for HaCat cells. Cells subjected to inflammatory stimulation with LPS, when treated with insect-derived chitosan, showed a significant reduction in the expression of the pro-inflammatory cytokines IL-6, IL-8, IL-1α, and TNF-α, compared to samples not treated with the biopolymer. This reduction was observed as early as 6 h after treatment, with optimal effects persisting up to 24 h, highlighting chitosan efficacy in modulating the LPS-induced inflammatory response.

Our results yielded a stronger anti-inflammatory activity for chitosan obtained through heterogeneous deacetylation than homogeneous ones. The different modulation effect between insect chitosan samples could be due to the different deacetylation conditions affecting the chemical and structural characteristics of the polymer; on the other hand, the possible influence of chitin bleaching on the anti-inflammatory potential of chitosan has to be ruled out.

The results obtained, however, demonstrated a correlation of anti-inflammatory activity with the insect biomass used rather than with the specific properties of chitosan. Indeed, no significant differences potentially ascribed to the variation in molecular weight (Mw) were found, but only to the deacetylation degree (DD) variation, considering the greater activity of the heterogeneous samples, with higher DD, compared to the homogeneous ones, with lower DD.^17,28^

However, concerning homogeneous deacetylated chitosan, unbleached samples effectively decreased cytokine expression for all of the cytokines studied, showing an anti-inflammatory effect. The difference in Mw between bleached and unbleached homogeneous chitosan can be assumed to influence the different anti-inflammatory activity.^28^

In the literature, there are no studies on the anti-inflammatory activity of chitosan from *H. illucens*, and for this reason, a direct comparison with existing published data was not possible.

There are several papers on the anti-inflammatory and pro-inflammatory properties of chitosan and its derivatives, as well as of the same polymer in different forms. Some authors, however, limited their focus to the evaluation of the influence of chitosan on cell proliferation, particularly fibroblasts and keratinocytes.^50,51^

Li et al.^12^ and Zhou et al.^68^ reported the immunomodulatory activity of low Mw chitosan derivatives, the oligosaccharides (COS). Pro-inflammatory cytokines (IL-1β, IL-6, and TNF-α) and anti-inflammatory cytokine (IL-2) levels in mouse models of osteoarthritis were evaluated, resulting in a reduction of serum expression of pro-inflammatory cytokines and an enhancement of anti-inflammatory activity.^12^

The concentration of 500 μg/mL was found to be capable of attenuating the expression levels and the release of inflammatory cytokines, also for COS,^69^ as well as for heterogeneous chitosan samples studied in this work. Particularly, unlike our results, the inhibitory activity of crustacean COS was found to be inversely proportional to the value of its Mw (lower Mw, higher activity).

Similarly, in the study performed by Kim et al.*,*^9^ the best anti-inflammatory effect was that of the biopolymer with low Mw and high DD. These data are in contrast to the results obtained in this work from *H. illucens* homogeneous chitosan; indeed, among homogeneous chitosan samples, the most effective anti-inflammatory activity was detected in an experiment in which the unbleached samples were used, i.e., those with the lowest DD and highest Mw. This, therefore, suggests a direct correlation between the anti-inflammatory activity of chitosan and the method of deacetylation but also suggests a possible connection with the purification processes (more strictly to the bleaching step, as reported before) of the analyzed sample. Other studies, such as Davydova et al.*,*^70^ have shown that the anti-inflammatory activity of chitosan is significant for both low Mw and high Mw (115 kDa and 5.2, respectively). Chitosan has proven effective in strongly inducing anti-inflammatory cytokine IL-10. The authors have thus demonstrated that the anti-inflammatory activity does not depend on the Mw of the polymer but on its molecular structure.^69^ In other studies, it is pointed out that chitosan, as the main film-forming constituent, has proven to be effective in inhibiting cytokines. Particularly, it caused a drastic reduction in pro-inflammatory cytokines and TNF-α in cells grown on chitosan films, along with an increase in anti-inflammatory cytokines IL-10 and TGF-β1.^71^ Also concerning chitosan nanosystems, the biopolymer, functionalized with alginate, has also proven to be effective in reducing cytokines and inflammatory chemokines induced by *Propionibacterium acnes*. Indeed, the biopolymer inhibited their production at the level of human keratinocytes and monocytes.^72^

The only papers in which the anti-inflammatory effect of insect chitosan was explored confirmed our findings. Kathami et al.^73^ and Li et al.,^74^ reported that chitosan from larvae, pupal exuviae and adults of *Tenebrio molitor* and from *Periplaneta americana* larvae, respectively, has an anti-inflammatory effect on macrophages, increasing the production of anti-inflammatory markers (TGFβ, IL-10 and IL-17) and reducing the pro-inflammatory ones (IL-1β and TNFα).

## Conclusions

Chitosan is a natural polymer that, due to its biological properties, can be employed in numerous fields of application, the most avant-garde of which are biomedical and pharmaceutical, directly related to human use. *H. illucens* is emerging as a sustainable alternative source for the production of the biopolymer, normally obtained from crustaceans, offering a more sustainable solution that meets the growing industrial demand. Its bioconverting ability makes the insect a promising resource for reducing environmental impact and improving sustainability in biopolymer production. Among chitosan properties, anti-inflammatory activity is one of the most prominent, due to the increase in chronic inflammatory diseases and the limitations of current drugs, such as severe side effects and long-term ineffectiveness. Inflammation is not only involved in autoimmune diseases, but also in cardiovascular diseases, diabetes, cancer, and neurodegenerative diseases, urgent issues to be handled.

In this work, the biological characterization of chitosan from larvae, pupal exuviae, and adults of *H. illucens* was deepened by carrying out studies on HaCat cells that were induced to an inflammatory response by bacterial LPS. Insect-chitosan samples, especially the heterogeneous deacetylated ones, showed a strong anti-inflammatory activity.

Hence, the biological characterization of chitosan from *H. illucens* points to its potential use (with even better results compared with the crustacean chitosan) in the same applications already established for the crustacean-derived polymer, including biomedical and pharmaceutical fields, in order to have beneficial effects on human health.

## Data Availability

The data sets used and/or analyzed during the current study are available from the corresponding author on reasonable request.

## Supporting Information Available

FTIR spectra of both unbleached and bleached, heterogeneous and homogeneous chitosan samples produced from *H. illucens* larvae, pupal exuviae and adults; commercial chitosan derived from crustaceans, taken as control, is also reported in the spectra; XRD spectra of both unbleached and bleached, heterogeneous and homogeneous chitosan samples produced from *H. illucens* larvae, pupal exuviae, and adults; commercial chitosan derived from crustaceans, taken as control, is also reported in the spectra (PDF)
