# Supplementary material for: Hermetia illucens-Derived Chitosan: A Promising Immunomodulatory Agent for Applications in Biomedical Fields
Source: Biomacromolecules. 2025 Apr 29;26(5):3224–33. doi: 10.1021/acs.biomac.5c00362 (PMC12076490; doi:10.1021/acs.biomac.5c00362)
Supplement: Supplementary file 1 — bm5c00362_si_001.pdf [file bm5c00362_si_001.pdf]

Hermetia illucens-derived chitosan: a promising immunomodulatory agent for application in biomedical fields

Alessandra Fusco[1,2]§, Anna Guarnieri[3]§, Carmen Scieuzo[3,4]*, Micaela Triunfo[3], Rosanna Salvia[3,4], Giovanna Donnarumma[2#], Patrizia Falabella[3,4#]*

[1] Department of Life Sciences, Health and Health Professions, Link Campus University, 00165, Rome, Italy
[2] Department of Experimental Medicine, University of Campania "Luigi Vanvitelli", Naples, NA 80138, Italy
[3]Department of Basic and Applied Sciences, University of Basilicata, Via dell'Ateneo Lucano 10, 85100 Potenza, Italy
[4] Spinoff XFlies s.r.l, University of Basilicata, Via dell'Ateneo Lucano 10, 85100 Potenza, Italy

§these authors contributed equally to this work

[#]these authors contributed equally to this work

*Corresponding authors: carmen.scieuzo@unibas.it; patrizia.falabella@unibas.it

Supporting Information for publication

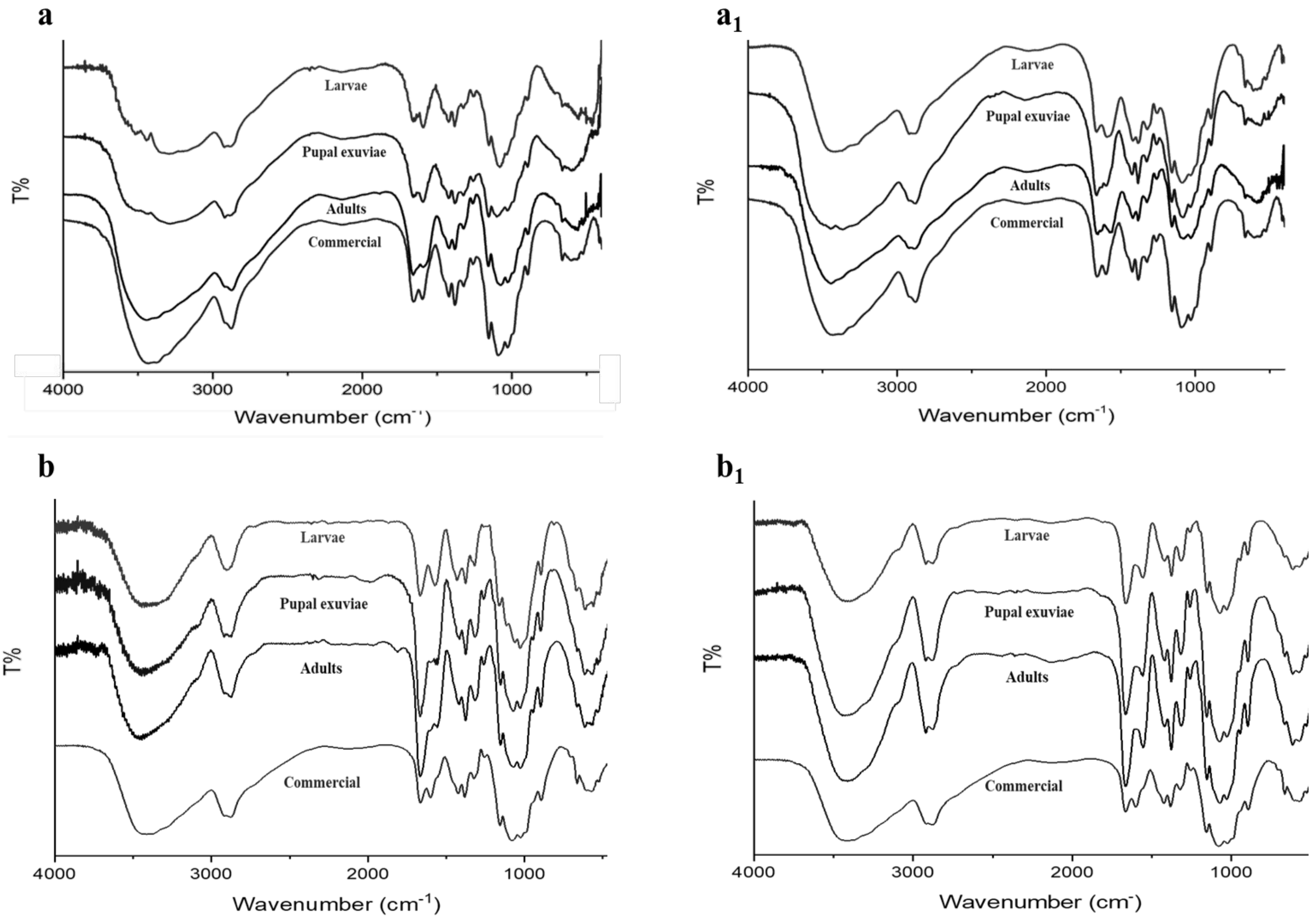

Figure S1. FTIR spectra of both unbleached (a, b) and bleached (a_1, b_1), heterogeneous (a, a_1) and homogeneous (b, b_1) chitosan samples produced from *H. illucens* larvae, pupal exuviae and adults. Commercial chitosan derived from crustaceans, taken as control, is also reported in the spectra.

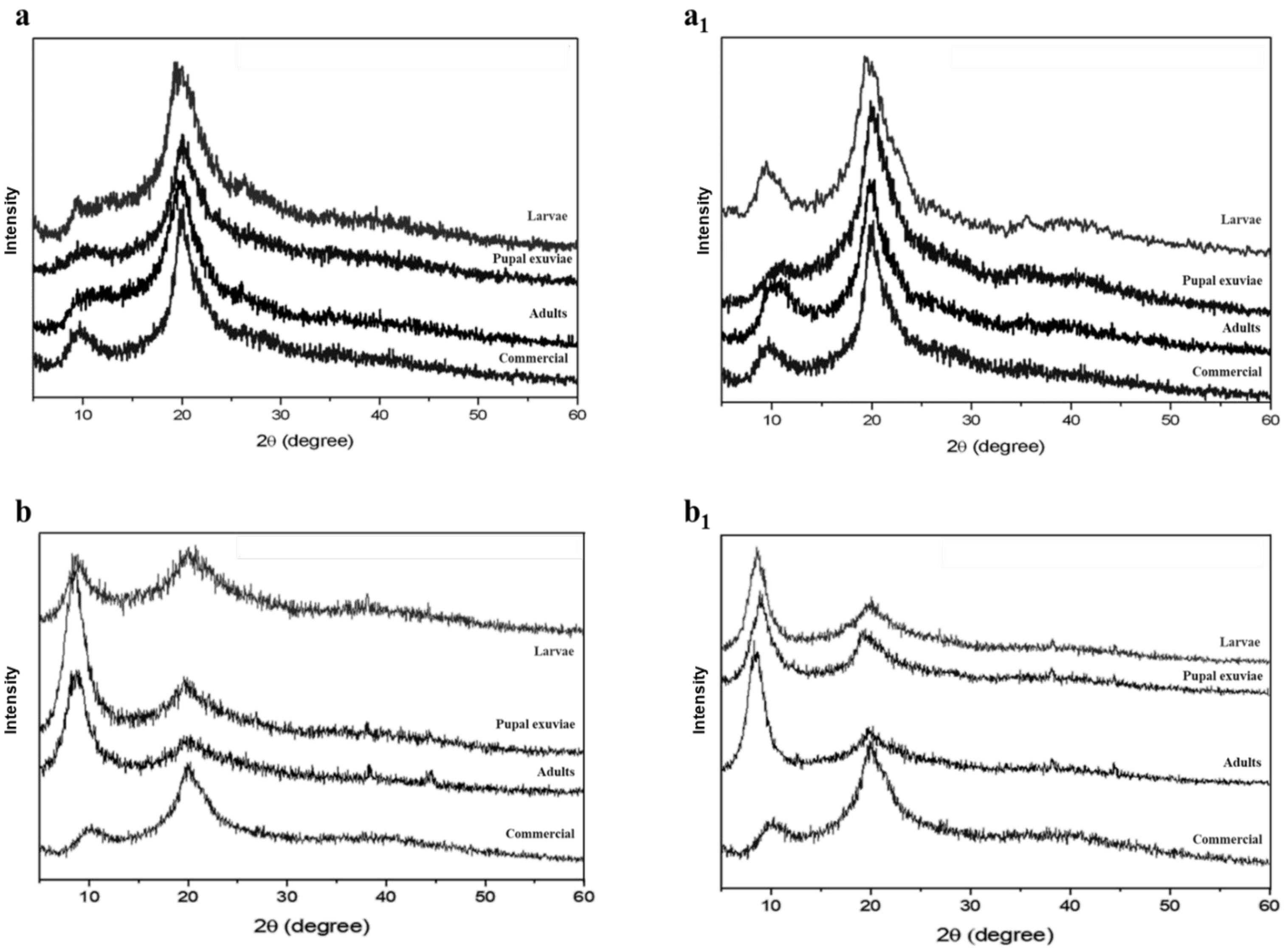

Figure S2. XRD spectra of both unbleached (a, b) and bleached (a_1, b_1), heterogeneous (a, a_1) and homogeneous (b, b_1) chitosan samples produced from *H. illucens* larvae, pupal exuviae and adults. Commercial chitosan derived from crustaceans, taken as control, is also reported in the spectra.
